# Acute Jaundice in a Six-year-old: An Unusual Presentation of Atypical Kawasaki Disease

**DOI:** 10.5811/cpcem.2019.12.45180

**Published:** 2020-02-24

**Authors:** William Bylund, Gregory J. Zarow, Daphne Morrison Ponce

**Affiliations:** * Naval Hospital Okinawa, Department of Emergency Medicine, Okinawa, Japan; † Combat Trauma Research Group, Naval Medical Center Portsmouth, Portsmouth, Virginia; ‡ The Emergency Statistician, Idyllwild, California; § University of Michigan, Department of Emergency Medicine, Ann Arbor, Michigan

## Abstract

Kawasaki disease (KD) is a rare vasculitis of childhood that is critical to recognize and treat due to associated morbidity and mortality. A six-year-old male presented to our emergency department (ED) afebrile but with reported recent fevers. Exam revealed jaundice and erythematous tongue with papules, and laboratory studies indicated a direct hyperbilirubinemia. Admitted for evaluation, he developed continuous fever, increasing maculopapular rash, and subsequent desquamation of hands and feet. He ultimately met criteria for incomplete KD, was treated with intravenous immunoglobulin, and avoided cardiac complications. This presentation of incomplete KD with hyperbilirubinemia is rare because the patient was afebrile at ED presentation.

## INTRODUCTION

Kawasaki disease (KD) is a systemic vasculitis affecting small and medium blood vessels. Originally characterized by Tomisaku Kawasaki in 1967, KD remains a challenging diagnosis based on clinical features. KD is relatively rare, with approximately 13.7 annual incidence per 100,000 in white children under five years of age. However, KD is a leading cause of acquired heart disease in North American and Japanese children, making KD identification an important area of interest for emergency providers.^1^

Classic KD is defined by a daily fever for greater than five days with at least four of five clinical criteria: bilateral bulbar conjunctival injection; oral mucosa involvement; peripheral edema; polymorphous rash; and cervical lymphadenopathy (with at least one > 1.5 centimeters [cm]). A diagnosis of incomplete KD can be made if two or three of the principal features are present in association with an echocardiogram showing coronary artery abnormalities or at least three of the following laboratory findings: albumin of three grams (g) per deciliter (dL) or less; anemia for patient’s age; platelet count 450,000 thousandths (K) per microliter (μL)or greater; white blood count (WBC) 15,000 K/μL or greater; elevation of alanine aminotransferase (ALT); and sterile pyuria greater than or equal to 10 white blood cells per high-powered field.^1^–^2^

Untreated, KD may progress to cardiac involvement in 25% of cases. This may include pericardial effusions, coronary artery aneurysms with rupture, cardiac ischemia, dysrhythmias, or even sudden death. Non-cardiac complications include peripheral vascular aneurysms or obstructions, nephritis, or sensorineural hearing loss.^1^–^4^ Unfortunately, KD is difficult to diagnose in the emergency department (ED) because no single diagnostic test can efficiently establish the diagnosis of KD, and fever with rash is a common complaint. The diagnosis of incomplete KD has been associated with a delay in intravenous immunoglobulin (IVIG) treatment.^1^ To demonstrate the complexity of KD diagnosis, the following case study describes an afebrile presentation of incomplete KD in the setting of acute jaundice.

## CASE REPORT

**Image 1:**
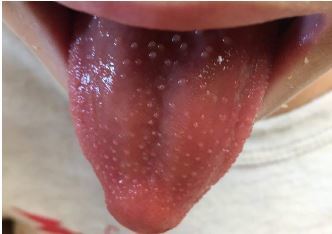
Erythematous tongue with papules, on day of emergency department presentation (day 4 of illness).

**Image 2:**
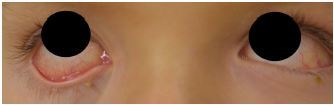
Jaundice, icteric sclera, and conjunctival injection, day four of illness.

An otherwise healthy six-year-old male presented to the ED on day four of illness with fevers, sore throat, and dysuria with dark urine. The patient saw his primary care physician on day one of illness with a temperature of 101.4 degrees Fahrenheit (F) and was diagnosed with a nonspecific viral illness, but his symptoms continued to worsen despite supportive care. In the days following the office visit, he had temperatures to 102° F daily, and developed jaundice, a maculopapular rash, and an erythematous tongue with papules (Images 1 and 2).

The patient presented at the ED without a fever in the setting of acetaminophen use at home (last dose unknown). History was obtained from the mother and patient, and they endorsed slight non-productive cough but otherwise denied associated symptoms. The patient was fully immunized and his past medical history was unremarkable. Initial ED exam revealed a well-appearing patient with temperature of 99.7° F, pulse 115 beats per minute, blood pressure 98/64 millimeters of mercury, respiratory rate 22 breaths per minute, oxygen saturation 97% on room air, and weight 19.7 kilograms. His head was atraumatic and normocephalic. However, his ear, nose, and throat exam revealed an erythematous tongue with flesh-colored papules (Image 1). His sclerae were icteric and injected without exudate, and the conjunctival injection spared the limbus (Image 2). No significant cervical lymphadenopathy was detected.

Cardiac auscultation revealed a regular rate and rhythm without murmurs, and the lungs were clear bilaterally. His abdomen was non-tender even to deep palpation. Skin exam was notable for diffuse jaundice and an erythematous maculopapular rash to the abdomen and face, sparing the palms and soles. He was awake and alert, following commands, and answering questions appropriately for his age.

Initial laboratory values were obtained for diagnosis. A complete blood count had a leukocytosis to 16.3 thousandths K/μL (normal range: 6.0–17.0 K/μL) with 86% neutrophils (35%–45%), and platelet count of 369 K/μL (210–490 K/μL). Total bilirubin was elevated at 5.4 milligrams per deciliter (mg/dL) (0.3–1.8 mg/dL), and conjugated bilirubin was 2.9 mg/dL (0.0–0.3 mg/dL). Hepatic panel revealed mild transaminitis (aspartate aminotransferase, alanine aminotransferase, and alkaline phosphatase, 59 units (U) per liter (L) (17–59U/L), 169 U/L (21–72U/L), and 425 U/L (150–380U/L), respectively. Urinalysis revealed trace protein, large bilirubin, no casts, 3–5 red blood cells with trace hemolysis, and 3–5 white blood cells. An initial pharyngeal rapid strep test was negative.

**CPC-EM Capsule**

What do we already know about this clinical entity?

There have been case reports of jaundice in Kawasaki disease (KD). However, since it is not part of the diagnostic criteria, it is often omitted from standard teaching.

What makes this presentation of disease reportable?

This is only the second case report in United States’ literature of KD presenting with acute jaundice.

What is the major learning point?

Emergency physicians should consider atypical KD in the setting of unexplained acute jaundice. Patients may be afebrile at time of presentation.

How might this improve emergency medicine practice?

Increased recognition of atypical KD can improve timely and accurate diagnosis, enabling earlier treatment and decreased complication rates.

The patient was admitted to the pediatric ward for antibiotics and serial exams, pending abdominal imaging, autoimmune testing, additional labs, viral serologies, and culture results. A comprehensive right upper quadrant ultrasound showed a normal appearing gallbladder and no intrahepatic or extrahepatic biliary ductal dilatation to suggest biliary obstruction. The chest radiograph noted possible right basilar airspace disease. However, given the lack of sputum, respiratory complaints, increased oxygen support, and the associated physical exam findings, pneumonia was considered to be inconsistent with the presentation.

Erythrocyte sedimentation rate (ESR) was elevated at 113 millimeters per hour mm/hr (0–10mm/hr) and C-reactive protein (CRP) was elevated at 3.5 milligrams (mg)/dL (less than 1.0mg/dL), consistent with possible KD.^1^ Gamma-glutamyltransferase was elevated at 241U/L (15–73 U/L). Throat cultures, antistreptolysin O, hepatitis B surface antigen, hepatitis B core antibody, hepatitis A immunoglobulin (Ig) M, hepatitis C antibody, cytolomegalo virus IgG/IgM, toxoplasmosis IgG/IgM, monospot, blood cultures, and leptospirosis testing were negative, making infectious or post-infectious etiologies less likely.

On day seven of illness (hospital day four), the patient developed bilateral cervical lymphadenopathy up to one centimeter, and his bilateral bulbar conjunctival injection worsened. Despite being afebrile at ED presentation, he continued to spike fevers in the hospital (until the sixth day of illness and third hospital day), despite administration of ibuprofen. During his admission, the patient had an elevated ALT, low albumin, thrombocytosis, and elevated WBC, thus meeting at least three supplemental criteria for KD, making incomplete KD the likely diagnosis. The case was discussed with a remote KD specialist, and the next day the patient was transferred to the intensive care unit for IVIG infusion. An echocardiogram was within normal limits, although limited by inability to assess the full length of the coronary arteries. The patient was initiated on aspirin 81 mg daily. He was discharged on low-dose aspirin with plans to obtain repeat outpatient echocardiograms.

**Image 3:**
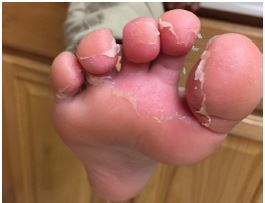
Desquamation of feet, day 13 of illness.

Two weeks after discharge, the patient was evaluated by a pediatric cardiologist. Desquamation of the hands and feet were noted (Image 3). Repeat formal echocardiogram was normal, without evidence of pericardial effusion or coronary dilation. Approximately two months after his illness, the majority of the patient’s symptoms had resolved and low-dose aspirin was discontinued. A repeat echocardiogram eight months after initial illness was normal, and a telephone follow-up at 10 months indicated that he continued to do well.

## DISCUSSION

KD is a rare but critical diagnosis for physicians to consider in ED settings. In our patient, many other diagnoses were considered but did not fit the clinical picture. The differential diagnosis included infectious causes, such as scarlet fever associated with hepatitis, viral hepatitis, or other viral syndrome, cholangitis, and non-infectious causes, such as KD, malignant or other biliary obstruction, drug reaction, or an autoimmune process, such as acute rheumatic fever. At ED presentation, the patient was afebrile and only had three primary criteria for the diagnosis of KD: conjunctivitis; strawberry tongue; and maculopapular truncal rash. An autoimmune process such as rheumatic fever was considered less likely as he had no arthralgias, and the rash was not the classic well-demarcated, semi-annular rings of erythema marginatum.

The recent fevers and elevated neutrophils increased the suspicion for infectious causes of unexplained jaundice. The patient was treated with penicillin for possible scarlet fever with associated hepatitis, pending streptococcal titers. Scarlet fever was considered a potential unifying diagnosis, as hepatitis with obstructive jaundice has been reported in cases of scarlet fever.^5^–^6^ Post-streptococcal glomerulonephritis was considered due to his tea-colored urine, but the laboratory values were inconsistent with the diagnosis. Viral etiologies such as mononucleosis were considered, but mononucleosis screen was negative. Cholecystitis with obstruction was considered, but the patient had no abdominal tenderness, and cholecystitis would be atypical in this age group.

As in other cases, the diagnosis of KD was difficult to make initially, but eventually the patient satisfied necessary criteria for incomplete KD. He had fever for greater than five days, conjunctival injection without exudate, oral mucosal involvement, a polymorphous rash with inguinal accentuation, cervical lymphadenopathy (although below the 1.5 cm criteria), and ultimately periungual desquamation. The ESR and CRP levels were elevated, which led physicians to look for supplemental laboratory criteria. The patient ultimately met four laboratory criteria: WBC greater than 15,000-K/μL; platelet count over 450,000-K/μL; an elevated ALT; and albumin less than 3.0 mg/dL.^1^

KD has previously been reported in patients with hepatitis and cholestatic jaundice. We identified nine previous case reports, with a total of 17 KD patients displaying jaundice.^7^–^15^ Although rarely reported, KD is the most common cause of febrile obstructive jaundice following viral hepatitis. A tertiary pediatric clinic chart review study reported that one in five jaundice cases were “caused” by KD.^13^ A recent case report described KD presenting with fevers and acute acalculous cholecystitis that responded to medical treatment (IVIG and aspirin) in lieu of urgent surgical intervention.^15^ Systemic vasculitis may underpin both KD and some cases of jaundice, and our case adds to the growing body of literature showing an association between KD and jaundice. Further empirical evidence of an association between KD and jaundice might justify adding elevated bilirubin to the supplemental criteria for incomplete KD.

## CONCLUSION

This case of incomplete KD was particularly challenging to diagnose because the patient was afebrile at time of presentation. This case also illustrates the difficulty in diagnosing incomplete KD when other more common diagnoses may initially be considered more likely, as sometimes only two or three of the clinical criteria are apparent at initial presentation.^13^–^14^ This patient had incomplete KD with associated conjugated bilirubinemia, adding to a growing body of literature showing an association between jaundice and KD. Unexplained jaundice with associated features should prompt physicians to consider KD. This case and others emphasize the importance for emergency physicians to retain a high level of suspicion for KD when a child presents with febrile or afebrile obstructive jaundice.^7^–^15^
